# Measures for Persons with Spinal Cord Injury to Monitor Their Transitions in Care, Health, Function, and Quality of Life Experiences and Needs: A Protocol for Co-Developing a Self-Evaluation Tool

**DOI:** 10.3390/healthcare12050527

**Published:** 2024-02-23

**Authors:** Katharina Kovacs Burns, Zahra Bhatia, Benveet Gill, Dalique van der Nest, Jason Knox, Magda Mouneimne, Taryn Buck, Rebecca Charbonneau, Kasey Aiello, Adalberto Loyola Sanchez, Rija Kamran, Olaleye Olayinka, Chester Ho

**Affiliations:** 1 Clinical Quality Metrics/Primary Data Support, Data & Analytics, Alberta Health Services, Edmonton, AB T5J 3E4, Canada; 2 School of Public Health, University of Alberta, Edmonton, AB T6G 1C9, Canada; 3 Spinal Cord Injury Alberta, Calgary, AB T2H 1H7, Canada; 4 ReYu Paralysis Recovery Centre, Edmonton, AB T5S 1G8, Canada; 5 Slave Lake Healthcare Centre (North Zone), Alberta Health Services, Slave Lake, AB T0G 2A2, Canada; 6 Tertiary Neurorehabilitation, Foothills Medical Centre, Calgary, AB T2N 2T9, Canada; 7 Glenrose Rehabilitation Hospital, Edmonton, AB T5G 0B7, Canada; 8 Physical Medicine and Rehabilitation, Foothills Medical Centre, Calgary, AB T2N 2T9, Canada; 9 Patient Liaison to Neurosciences, Foothills Medical Centre, Calgary, AB T2N 2T9, Canada; 10 Division of Physical Medicine & Rehabilitation, Faculty of Medicine & Dentistry, University of Alberta, Edmonton, AB T6G 2S2, Canada; 11 Department of Clinical Neurosciences, Glenrose Rehabilitation Hospital, Edmonton, AB T5G 0B7, Canada; 12 Rehabilitation Sciences, Faculty of Rehabilitation Medicine, University of Alberta, Edmonton, AB T2N 1N4, Canada

**Keywords:** protocol, self-evaluation measures, persons with spinal cord injury, transitions in care, health changes, function changes, quality of life, mixed methods, modified Delphi, tool co-development

## Abstract

Evaluating the experiences of persons with spinal cord injury (PwSCI) regarding their transitions in care and changes in health, function, and quality of life is complex, fragmented, and involves multiple tools and measures. A staged protocol was implemented with PwSCI and relevant expert stakeholders initially exploring and selecting existing measures or tools through a modified Delphi process, followed by choosing one of two options. The options were to either support the use of the 10 selected tools from the Delphi method or to co-develop one unique condensed tool with relevant measures to evaluate all four domains. The stakeholders chose to co-develop one tool to be used by persons with SCI to monitor their transition experiences across settings and care providers. This includes any issues with care or support they needed to address at the time of discharge from acute care or rehabilitation and in the community at 3, 6, and 12 months or longer post-discharge. Once developed, the tool was made available online for the final stage of the protocol, which proposes that the tool be reliability tested prior to its launch, followed by validation testing by PwSCI.

## 1. Introduction

As more is known through studies and personal stories regarding the complex nature of spinal cord injuries (SCI), both traumatic and non-traumatic, more is understood regarding the long-term impacts on persons with SCI (PwSCI) and their physical, psychological, social, and economic wellbeing [1,2,3,4,5,6,7]. Physically, SCI permanently transforms an individual’s life through impacts on motor, sensory, and autonomic body systems [8], resulting in different impairments in mobility, bowel and bladder functions, sensation, and sexual function, as well as secondary health conditions such as pain, pressure ulcers, and urinary tract infections [3]. Regarding the psychological well-being of PwSCI, studies have shown higher rates of psychological disorders including depression [5,6,9]. Socially, studies have shown that the relationships of PwSCI with family and friends, as well as participation or reintegration within the community including returning to work or school, are also impacted [10,11]. In addition, PwSCI face other structural, infrastructural, and environmental challenges, such as accessibility to buildings or facilities, services or programs including leisure/recreation, and transportation [12,13,14]. The economic impacts of SCI are not only associated with employability or returning to work but also with accessing specific third-party disability funding sources to support daily needs, out-of-pocket costs, and equipment including wheelchairs, as well as living accommodation renovations [15].

Although these studies suggest there are common physical, psychological, social, and other well-being impacts for all or most PwSCI, it is difficult to generalize about their individual personal experiences and needs. There are many factors that need to be considered, including personal attributes, capacity to adjust, and support network(s) [13]. Individuals also face different challenges at different times during their acute or rehabilitation care, discharge process, and community reintegration. Community integration includes consideration of the person’s home or community context and access to available care and support within these settings. A person’s access is, in turn, determined by the community‘s capacity to provide resources as well as make accessible much-needed health and social support, services, and programs for PwSCI [16]. This includes PwSCI having access to ongoing rehabilitation services and knowledgeable and skilled healthcare professionals, which some suggest are essential [17]. These many factors, along with the complex nature and impact of SCI, determine the level of life-long care, support needs, experiences, and expectations of PwSCI.

Various measurement tools have been tested and some validated by PwSCI to assess the impact of SCI on their lives, including their experiences and/or needs. For example, different aspects of health, including medical, have been measured using such tools as the International Standards for Neurological Classification of Spinal Cord Injury–ASIA Impairment Scale (ISNCSCI–ASIA) [18]; Short Form Physical and Mental Health Scale-12, -20, or -36 (SF-12, SF-20, SF-36) [19]; or Patient Health Questionnaire-9 (PHQ-9) [20]. Function, including mobility, was shown to be measured by tools such as the Spinal Cord Independence Measure Version III (SCIM III) [21]; EuroQol 5 Dimensions 5 Levels questionnaire (EQ-5D-5L) [22]; or Craig Handicap Assessment and Reporting Technique (CHART) [23]. Of the many tools used to measure the quality of life (QOL), some included the WHO Quality of Life Assessment (WHOQOL-BREF) [24] and SCI Quality of Life Ability Assessment (SCI-QOL-A) [25]. All of these or other tools were usually applied for PwSCI at specific points of time while they were in acute or rehabilitation care settings or in community care following their discharge. Some of these assessment tools have also been used to track changes in health, function, and QOL of PwSCI over time, or as their health and psychosocial care needs change over time.

Unfortunately, the information around any measured experiences of PwSCI related to their care, services, support, and resulting lives, as well as their different care and personal needs, is fragmented. Very few studies explored and described the complete or comprehensive longer-term journeys of PwSCI. This included their lifelong changes in health status, function, and QOL, as well as care needs [26], across the continuum of care from acute to rehabilitation to community, as well as multiple transitions across care settings and care providers [1,16]. Rather, most studies appear more focused on such areas as the array of health or individualized care needs [26,27], or one of the setting contexts of acute, rehabilitation, discharge [28], or community care or follow-up [15,29,30,31]. Various challenges, problems, or gaps experienced by PwSCI inhibiting them from fulfilling their needs have been shown to impact their QOL; increase their chances of morbidity, adverse events, hospital readmissions, emergency room visits; and/or decrease their overall satisfaction with life’s experiences [32]. Very few studies explored the integrated needs and experiences of PwSCI with their family caregivers or with various health care providers [1].

In Alberta, Canada, a diverse study team consisting of PwSCI, clinicians, other care providers, health care administrators/decision makers, community partners, and researchers collaborated to examine transitions in care (TiC) issues experienced by PwSCI and explore some solutions as a provincial priority, recognizing that similar issues exist across Canada [16,17]. The study called CONnecting and Coordinating an Enhanced Network for TRansitions in Care (CONCENTRIC) aimed to design, implement, and evaluate an Alberta SCI TiC model integrated into a provincial “hub and spokes system” (i.e., hubs were identified as large urban centers with tertiary and other care/supports in place, while spokes were the smaller more rural and/or community centers with some or minimal care/support). As part of this study, PwSCI and SCI stakeholders expressed the need for evaluation tools to measure: (1) what PwSCI experienced regarding their care and transition journey from acute or rehabilitation to community settings, including changes in their health, function, and QOL; and (2) what care, resources, and support were accessed or needed by PwSCI at various times during their transition journey (i.e., at discharge from acute or rehabilitation care to the community, and follow-up at three, six or twelve months post-discharge). This evaluation aspect of the study was of interest not only in Alberta but across Canada, as indicated by diverse cross-Canada stakeholders who were members of the national CONCENTRIC Advisory Committee.

In this paper, we describe the protocol including the process, challenges identified, and outcomes of exploring and co-selecting existing measures and/or co-developing new ones that would be appropriate for a tool to be used by or with PwSCI to evaluate or monitor their TiC journey. The goal was to have a set of measures or tool with a dual function. The first function was focused on PwSCI being able to monitor or track their needs, gaps, and changes over time related to their TiC, health, function, and QOL. The second function was to inform and have care providers review and address identified needs and gaps of PwSCI related to their transition journey and changes in health, function, and QOL. Specifically, the tool was to follow the transition experiences of PwSCI to address any identified needs and gaps at the time of their discharge from acute or rehabilitation settings in tertiary centers and over different periods of time while in their home or community settings.

## 2. Methods

### 2.1. Study Setting and Context

The study setting was in Alberta, Canada, and utilized the mentioned “provincial hub and spokes system” involving the two specialized tertiary care centers in Edmonton and Calgary (the hub sites) and two community sites in Slave Lake and Lethbridge respectively (the spoke sites). The hub and spoke system approach represents the TiC pathway for persons with a recently diagnosed SCI receiving care from acute inpatient rehabilitation settings and discharged or transitioned into a community setting. In our situation, the PwSCI would receive initial acute and rehabilitative care in Edmonton and Calgary and be discharged into respective rural settings (i.e., Slave Lake from Edmonton and Lethbridge from Calgary).

Supplementary Figure S1 shows the geographical context of the hub sites in relation to the spoke sites [33]. Alberta has a population of 4.7 million people [34], with 2.5 million in Calgary and 1.1 million in Edmonton (see Figure S1, shown in File S1).

Our work to identify appropriate measures or tools as part of the CONCENTRIC study was based on the results of interviews conducted with diverse Alberta stakeholders (analyzed and reported in other CONCENTRIC study papers). From these latter results, six outcome domains were identified as being essential. They were:

(1) Transitions in Care or TiC (i.e., knowledge translation from stakeholders to patients/persons with SCI and patient awareness of resources after discharge from acute/rehab care);
(2) Changes in Health (both physical and mental health);
(3) Changes in Function (instrumental activities of daily living or IADL, self-management and activities of daily living or ADLs, and community participation and reintegration);
(4) Changes in QOL (all aspects of QOL);
(5) Changes in System Stakeholder’s Experience (patient-reported experience measures or PREMS, which measure quality of care during intervention; clinician experience with care delivery and health system; and caregiver/family or support person experience with care and health system);
(6) Improvements in Preventable Complication Rates (pressure injuries and urinary tract infections).

One of the goals of the CONCENTRIC study was to have self-evaluation tools for PwSCI to examine or measure their experiences. This includes evaluating their care, support, needs, and challenges with their transition from acute or rehabilitation settings to the community, as well as with changes in their health, function, and QOL over time. Therefore, the first four outcome domains were most appropriate to explore for this goal. The other two domains focusing on other system stakeholders and preventable complication rates were further explored and reported separately in other work of CONCENTRIC.

### 2.2. Evaluation Working Group

An Evaluation Working Group was established with clear terms of reference. This was to guide the process and decisions and to provide the most equitable opportunity for diverse SCI stakeholders to be involved in the work of exploring and/or developing the evaluation measures or tools for the four CONCENTRIC outcome domains. An email to the broader SCI community included an announcement about our planned work with details about the Evaluation Working Group and an invitation for interested individuals to contact us. Our terms of reference for the group called for a minimum of 15 diverse stakeholders with different SCI experiences or expertise, including those with lived experience. It was important to have an appropriate representation of the different SCI stakeholders and still be manageable in terms of size. Sixteen stakeholders formed this working group, consisting of three PwSCI, two physicians/clinicians, four allied health professionals, three community service providers, and four others from across the continuum of acute care, rehabilitation, and discharge/transition services. Four CONCENTRIC team members provided facilitation and operational support for the group.

### 2.3. Protocol Design and Approach

A staged protocol design was implemented, with each consecutive stage reliant on the findings of previously completed stages. Multiple methods were used as part of this design, with each stage having its own approach and methods. The aim of each stage was to provide the best opportunity for the working group stakeholders to view and narrow the scope of tools and measures for each domain. The group started with a comprehensive list or inventory of existing currently used tools and measures. The aim was to narrow this list to one that stakeholders viewed as comprising the most commonly used tools with informative measures that more accurately assessed the experiences of persons with SCI regarding the four domains (i.e., TiC and changes in health, function, and QOL) and across them.

**Stage 1: Identifying Tools Used for Measuring Four Outcomes with PwSCI**

Initially, a comprehensive search was conducted of the literature as well as various clinical or other inventories of tools or measures used by different stakeholders for assessing experiences of PwSCI or other chronic disabling conditions. We targeted those tools specifically regarding the four domains. Inclusion criteria were used to guide the search of the literature and other sources for tools or measures that could be considered as part of our review. Tools/measures could meet one, many, or all of the criteria to be considered. The inclusion criteria are shown in Table 1.

A review of gathered tools or measures followed and was based on more refined inclusion and exclusion criteria, beginning with eliminating any tools/measures that had not been used to measure the experiences of persons with traumatic and non-traumatic spinal cord injuries. The remaining tools/measures were further screened by stakeholders using more applied or practice-driven criteria such as those being currently used in practice settings, easy to apply, and reasonably accurate in reflecting the experiences and outcomes of PwSCI regarding each of the four domains. In addition, the tools/measures should have been completed with or by PwSCI during one or more times through their journey including in-patient rehabilitation, discharge from acute care or rehabilitation, and /or in various community care/clinic or other settings.

**Stage 2: Confirming Most Appropriate Tools Used For Measuring the Four Domains**

Based on analysis of the tool or measure review with the resulting list of selected tools or measures from Stage 1, the methods or approaches in Stage 2 included consultations with as many of the 90 invited diverse stakeholders as possible. It was important to include these 90 stakeholders, as they had identified or self-identified as being actively involved in various SCI care or support teams or networks from across Alberta (i.e., PwSCI, clinicians, other care providers including allied health, community service providers, researchers, managers/leaders, others). These 90 stakeholders formed our main network of SCI experts and, therefore, contacts in Alberta. Through these consultations, the goal was to have these SCI experts further narrow the selection of tools or measures used with PwSCI regarding their experiences and outcomes for each of the four domains. 

A modified virtual web-based Delphi technique [35] was determined to be the best approach for this process in Stage 2, which included having as many of the 90 invited expert stakeholders as possible participate in the Delphi Rounds and achieve consensus on the tools or specific measures needed to appropriately assess the experiences and outcomes of PwSCI for each of the four domains and aid in determining areas in which they needed further interventions or support [36]. With COVID-19 pandemic restrictions in place, we were forced to adapt the standard Delphi protocol. As a result, a modified Delphi approach was implemented using virtual connection and web-based online survey tools with stakeholders. Two modified Delphi Rounds were proposed: first, a priori, to achieve having diverse SCI expert stakeholders including PwSCI screen the various tools using one set of questions; and second, having these expert stakeholders identify through a set of more refined questions which of the selected tools were most appropriate and preferred for self-evaluation by PwSCI regarding their experiences with transitions in care and changes in their health, function, and QOL.

In Delphi Round 1, 90 diverse stakeholders were invited to be involved. The intent was to have as many of these diverse stakeholders as possible participate in the Delphi Rounds to eliminate some of the bias in the tool screening and selection. As numbers of participants decrease, there is a greater possibility for one or two groups of stakeholders to dominate as participants. We heard from different groups of stakeholders that they had preferences for using specific tools or measures with PwSCI. In order to mitigate this potential bias, we needed to have larger numbers of diverse stakeholders participate. An email was sent to all identified stakeholders containing information on the CONCENTRIC study, and a request to complete an online survey developed in a secure Alberta Health Services’ Research Electronic Data Capture (REDCap) site, following their review of the tools identified for each domain/sub-domain. Links to each tool were provided along with summary tables and psychometric properties, so respondents could review each tool in as much detail as they wanted to. The online survey was anonymous and asked respondents to review each tool and select one response that would indicate if they agreed to ‘keep—currently used/useful’, ‘keep but modify/adapt’, ‘discard’, or were ‘not sure’. If participants chose to complete the tool review and respond to the survey, their consent was implied. Adequate time for stakeholders to review and respond to the survey was determined to be four weeks, with reminders at two weeks and, if needed, an additional amount of time could be considered. This amount of time was intentional so as to avoid making this review a burden to respondents. Although Delphi Round decisions have no set consensus rate, an average of 75% agreement on tool/item selection was viewed as a reasonable level [37]. This also required consideration of the overall stakeholder response rate and diversity of respondents.

As per acceptable Delphi methods [35,36,37], there are various approaches to managing large numbers of stakeholders in the different Delphi Rounds. If the stakeholder group is extremely large to work with, a more purposive ‘expert’ panel of diverse participants can be selected for Delphi Round 2. From the 90 stakeholders invited to take part in Round 1, a panel of experts was selected based on their expertise in SCI and familiarity with using some or many of the selected tools for measuring the experiences or outcomes of PwSCI regarding the four domains. Based on a review with stakeholders, this narrowed the panel sample to 40 diverse experts (from across acute care, rehabilitation, clinical, and community settings, as well as PwSCI). Bias would be minimized by the diversity and balance of stakeholders selected from Rounds 1 to 2. The 40 experts were sent the email invite for Delphi Round 2 along with tables of tools and a more focused online REDCap survey asking them to select which tools identified during Delphi Round 1 were most appropriate and used for measuring the experiences of PwSCI across the four domains. They were also asked to provide any further comments or reasons for selecting the tools or measures they did.

A final list of selected tools from this stage was shared with the Evaluation Working Group for their review and discussion as part of Stage 3.

**Stage 3: Discussing Options Regarding Tools Selected**

Discussion of the Evaluation Working Group regarding the final inventory of selected tools from Stage 2 was the focus of work in Stage 3. The working group was presented with two options to discuss: (1) presenting the final inventory of selected tools for the four domains, with reference to the purpose, significance, and use of the tools in practice; or (2) exploring the co-development of one condensed tool of measures adapted from the multiple tools selected in Stage 2 and designed for PwSCI to self-evaluate all four domains. 

The decision regarding the options rested on discussions regarding:

- what the purpose of the tool/s was or how the tool/s could be used and in what context;
- how the tool/s met the criteria of the CONCENTRIC study regarding evaluation of experiences and outcomes related to each of the four domains;
- who the tool/s was/were intended for and who would use the tool/s;
- how the data or findings from the tool/s would be shared with appropriate individuals and used to address the care or other needs of PwSCI;
- how reasonable it was to assume that the selected tool/s measuring the four domains would be used by the appropriate stakeholders to measure and guide the journey of PwSCI.

**Figure 1:**
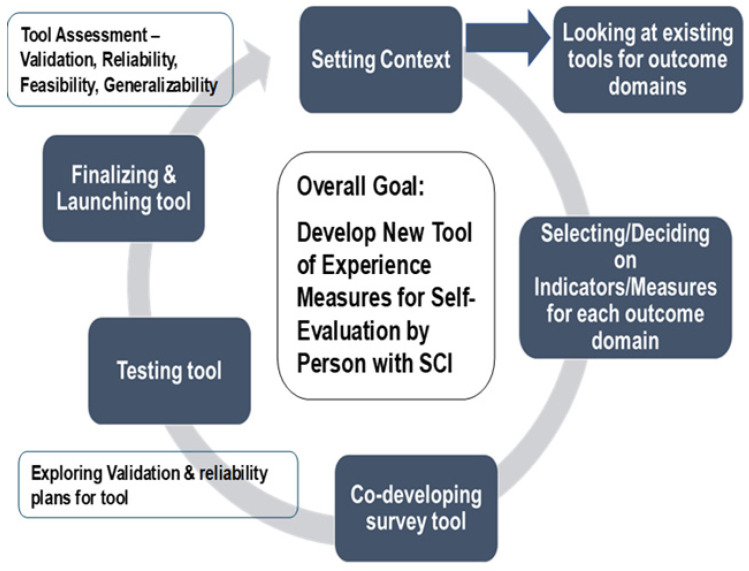
Process for Option 2—Tool Development Working Group reviewing and selecting measures for transitions in care and changes in health, function, and QOL domains with the goal of developing one tool of measures to be used for self-evaluation by persons with spinal cord injury (PwSCI).

As part of the protocol, a Tool Development Working Group would be established if the second option was chosen. This group would follow a process for reviewing the selected tools from the Stage 2 Delphi process and deciding on measures from each tool that would best gauge the experiences and outcomes of PwSCI regarding the four domains. This latter working group would follow the process shown in Figure 1 for developing one tool and be guided by the principles of co-development (i.e., all stakeholders would be provided with the information and directions to ensure they knew and were comfortable with the process and goals; all participants have an equal say in the discussion and decisions; decisions are based on 75% or greater agreement/consensus).

**Stage 4—Preparing Tool/s of Selected Measures for Piloting and Validity/Reliability Testing**

Regardless of the option chosen or the tool or tools selected to be used for self-evaluation by PwSCI regarding their experiences and outcomes for the four domains, the plan was to prepare or set things in motion for Stage 4, which was intended to have the tool or tools implemented for validity and reliability testing. This stage would commence with pilot testing of the tool or tools with a purposive sampling of 5 to 10 persons with SCI. This pilot testing would determine what tool of measures helped PwSCI best evaluate their experiences and outcomes regarding each domain specifically for the aspects that worked well for them and the aspects that still needed to be addressed.

Results from the pilot testing of the tools would guide further discussions by the working group as to the next steps in determining (1) preparation for reliability and validity testing of measures within the one or more tool/s for traumatic and non-traumatic populations of SCI; and (2) determining when, how, or where the one or more tool/s would be disseminated, used, or followed up as part of joint discussions between PwSCI and care providers. Once a clear path for the tool/s was developed and approved by stakeholders, preparation would begin to implement and track the results of both steps.

Since this paper describes the protocol for the final selection and/or development of one or more appropriate tools for measuring and monitoring the experiences of PwSCI across all four domains, a full description of the implementation of Stage 4 methods, analysis, and results has not been included in this paper. Another manuscript provides the latter details in a follow-up paper.

## 3. Results

**Stage 1—Identifying Tools Used for Measuring Four Outcome Domains with PwSCI**

The results for the initial comprehensive search of the literature and other sources resulted in 84 tools, which were further narrowed to 32 different tools based on the inclusion criteria applied. These included the review of psychometric properties indicating whether or not the tools had been tested and validated for use with the SCI population. Some tools were applicable to more than one domain (e.g., Spinal Cord Independence Measure or SCIMIII and Canadian Occupational Performance Measure or COPM). Table 2 provides the list of tools identified from the literature and practice guides for each of the four outcome domains.

**Stage 2—Confirming Most Appropriate Tools Used to Measure the Four Domains**

**Figure 2:**
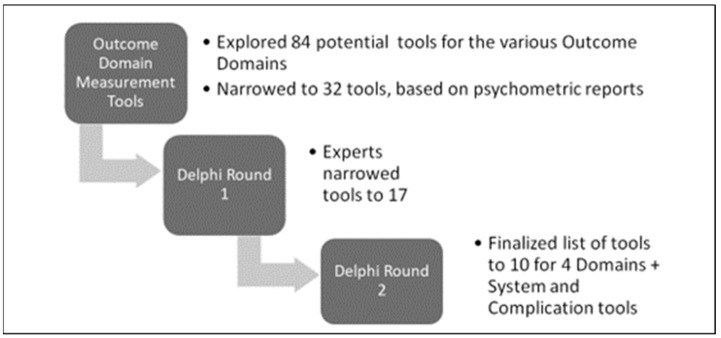
Delphi Rounds narrowing the selection of tools for persons with SCI.

Through the modified Delphi Rounds, a further narrowed selection of tools for each domain was achieved, as summarized in Figure 2.

In Delphi Round 1, 68 of the invited 90 stakeholders reviewed the 32 tools and screened them using the information provided about the tool, including any psychometric testing or validation reports and questions posed in the online survey or directly during virtual planned meetings. Only tools screened by the two categories as ‘keep—currently used/useful’ and ‘keep but modify’ were selected in this round. Respondents selected 17 of the 32 tools across the four domains, with some overlapping several domains. 

In Delphi Round 2, 40 invited diverse SCI experts (from across acute care, rehabilitation, clinical, and community settings and including PwSCI) completed the survey and/or took part in planned virtual discussions (75% response rate). Of the 17 tools identified in Delphi Round 1, 10 were selected in Round 2. The final list and profile of each of the 10 tools are shown in Table 3, including the number of items in each tool, measurement constructs, and validation by the SCI population.

**Stage 3—Discussing Options Regarding Selected Tools**

When the Evaluation Working Group was presented with the final selection of 10 tools for each of the four outcome domains and across the domains, they were able to discuss the options. Members of the working group were asked to anonymously respond to a poll as part of one of the virtual group meetings, indicating their agreement to pursue the co-development of one condensed tool or their concerns with this approach. All members were asked to respond. They unanimously decided to pursue the co-development of one condensed tool. The group felt that having one tool would align with the proposed dual role—i.e., be more reasonable for PwSCI to complete as a self-evaluation of their experiences and be useful as a guide in their discussions with their various care providers regarding what has worked well or where further support or care were needed. It was also determined that an ideal tool could be used at or shortly after the person is discharged from acute or rehabilitative care, at three or six months post discharge, one year post discharge, and greater than one year post discharge. These times mark key milestones for persons with SCI returning to and becoming established in the community and having access to appropriate or needed follow-up care and support after their discharge from acute or rehabilitation settings.

A sub-group of the Evaluation Working Group, called the Tool Development Working Group, was established to further explore the pros and cons regarding the option of developing one tool based on additional stakeholder discussions. This group, therefore, explored the purpose or intention of having one condensed tool, including the advantages for PwSCI to use one tool versus 10 to monitor the four domains. The group also explored the significance of having select key measures and questions in one tool for each of the four domains rather than over 20 measures and hundreds of questions, as found in the 10 tools. One tool with selected measures and questions would still capture what was working well for PwSCI and what needed more attention in the way of care or support. It was also significant to have one tool co-developed by PwSCI along with other stakeholders that would be used by PwSCI to monitor/track changes in their health, function, and QOL post discharge from acute care/rehabilitation into the community. The tool they developed could identify areas of care, support, and follow-up that would be needed to address the gaps in their health/well-being, function, and quality of life. PwSCI could share and discuss this information during their visits with various health care providers and community support they connected with for care and support. 

### Process for Tool Development

**Figure 3:**
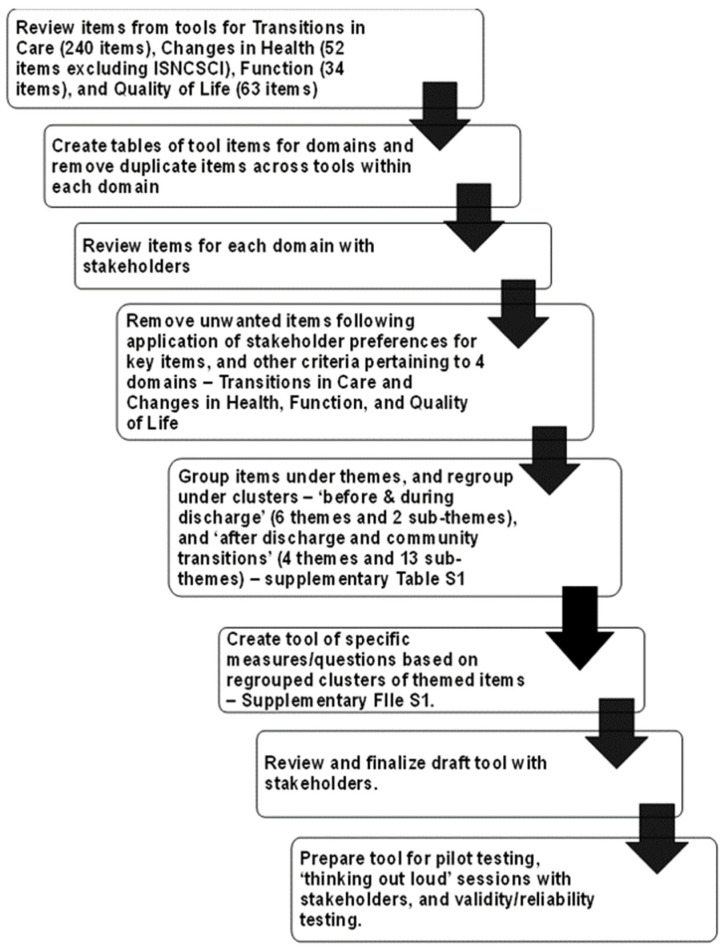
Sequential steps applied in screening and selecting measurement items for each outcome/domain, and tool development for persons with SCI.

The Tool Development Working Group applied a sequential stepped screening process with the prepared tables of measures/items for the various tools selected through Delphi Round 2 for each of the four outcome domains, as shown in Figure 3. 

As each table was screened, common items/measures within each domain were clustered under themes, while unique or single items were singled out. Further screening captured what stakeholders viewed as significant items for persons with SCI to measure for each domain and across the domains. The themes and their related items were mapped under two main categories—‘Before/During Discharge’ and ‘After Discharge—Self and Home/Community Transition’, as shown in Supplementary Table S1.

Once the Tool Development Working Group approved the themes and measures, questions were pulled from the 10 tools and reworded and/or adapted to one tool with a common Likert scale that most appropriately fit throughout. Choosing one Likert scale was viewed as essential to keep things simple and easy for those responding to the questions in the tool. Scores were not assigned to scales at this time but were viewed as something to consider if seen as relevant. Likewise, color coding of scales was not used to measure the level of urgency of the need for various items, but this too was discussed as a potential way to flag those items needing more timely attention as PwSCI discussed their situations with their care or service providers.

The final draft tool developed and approved by the working group to be pilot-tested with PwSCI is attached as Supplementary File S1.

**Stage 4—Preparing Tool of Selected Measures for Piloting and Reliability/Validity Testing**

The final tool version was loaded into REDCap, a secure online survey platform. This version was prepared for the next phase of tool testing and development, which included pilot testing it with 5 to 10 PwSCI, along with the cognitive reliability testing or ‘think aloud’ focus groups/discussions with PwSCI as well as other diverse stakeholders. The intent of these focus groups was to discuss the tool, its benefits, utility, and any challenges with it, as well as aspects of measures or questions concerning validity and reliability. Once this was complete, the tool was refined and launched with persons with SCI to obtain about 100–150 completed surveys for the validation testing. The end goal was to have a tool that would demonstrate reliability and validity for the SCI population. This latter stage of work and the results are reported in another paper. 

## 4. Discussion

In this paper, we described the protocol and outcomes of working with key SCI stakeholders, including PwSCI, to explore and make decisions about what tools and/or specific measures were most appropriate and relevant for monitoring the TiC journey of PwSCI and changes in their health, function, and QOL. The protocol design aligned with the participatory approach adopted for the CONCENTRIC study—we applied a co-design approach throughout, with stakeholders and particularly PwSCI co-selecting or co-developing appropriate measurement items or questions. Our stakeholders, as also shown in the literature, identified the need to identify the physical, mental, social, and quality of life needs of PwSCI along with barriers or factors associated with not having their needs met [16,26,38,39,40]. This was also true for identification of the experiences, outcomes, and needs of PwSCI regarding their transition journey including related changes and impacts they experienced as a result of the type of transition journey they had [41,42,43]. We did not find one tool in the literature that covered all domains but noted with interest the number of different tools that various studies either developed, tested, or identified, some of which are listed in Table 1. Hence, we were interested in exploring and examining the specific items/measures in various tools to more appropriately align with the experiences of PwSCI related to their transition journey and changes in their health (physical and mental), function, and QOL. 

Similar to our study, others described processes for identifying measurement domains and indicators specifically for PwSCI. For example, a few studies focused on processes for identifying measures related to rehabilitation and community-based experiences [16,30,43,44]. Processes varied in these studies, but the unique aspect of our study is that we used a rigorous staged approach in the protocol to guide the co-design work to explore and select/develop our tool/measures, with each subsequent stage being dependent on key actions or findings from the previous stage. This was part of our first two stages when key stakeholders had the opportunity to review and select key tools being used or seen as possible useful tools for measuring our four domains. It was also in these early stages that they were struck by the fact that so many tools existed, and many of these tools were redundant or had redundant measures. They questioned whether it was reasonable to have PwSCI along with their care providers use all of the 10 tools they selected after the Delphi Round 2 decisions. As a result, they identified the need to develop one tool that would encompass key measurement aspects to assess the experiences of PwSCI regarding their care transitions and changes in health, function, and QOL. 

The Tool Development Working Group justified the development of one tool for several reasons. This tool could be completed by PwSCI independently or with family/support persons or care providers, and the content would promote self-management but also identify what care/services they needed to seek out. This was identified by Jeyathevan et al. [43] in the development of the self-management indicators for PwSCI. The intent of the latter tool was to help facilitate conversations between PwSCI and various care providers regarding their self-management priorities and learning needs. Similarly, the one tool proposed for this initiative would serve as a point-in-time snapshot (i.e., at the time of discharge or 3, 6,12, or more months post-discharge). It could capture high-level indications of care and support needs that PwSCI had addressed or were satisfied with, and which ones were still in need of being further explored and addressed through their various care providers. Because each PwSCI would have a different or unique journey with transitions and unique health, function, and QOL experiences, this tool would assist or guide PwSCI to better understand their experiences and needs and, as a result, seek out or have more detailed conversations with their care providers. Stakeholders also felt it was important to have a tool that could be used at several points in time at discharge or post-discharge so that the care or support needs of PwSCI could be appropriately addressed and/or reviewed and updated between those points in time. This tool was the first such tool to address what the stakeholders felt was needed—i.e., measures across four outcome domains and at different transition journey points in time. However, it should be noted that the tool developed through our work was not intended to replace the 10 tools selected by stakeholders or even other tools noted in the literature. There was still an understanding that those 10 tools or others were designed to be used as needed by care providers to seek more in-depth understanding about the experiences of PwSCI that were identified as issues or gaps in their care transitions, health, function, and QOL. In addition, the developed tool could parallel others like the self-management indicators tool developed around rehabilitation [43] or complement the SCI Community Survey (SCICS) [16] or the community follow-up questionnaires that are part of registries such as the Rick Hansen Spinal Cord Injury Registry (RHSCIR) [45]. In addition, the co-designed tool could be considered to increase participation in longitudinal registries, such as the RHSCIR Community Follow-up Questionnaire (RHSCIR CFQ). As participants of the Edmonton RHSCIR CFQ were also part of our CONCENTRIC tool co-design process, they contributed to choosing the appropriate measures for a shorter tool and harmonized response format. They clearly reported that the current RHSCIR CFQ, which contained outcome measures for the same four domains and others, was too long and burdensome, thereby negatively affecting the will of people to complete it. 

Challenges were identified with developing and having one tool. Selecting and deciding on what items or measures were most important to capture in the tool took more commitment and time and much debate or discussion among the stakeholders but particularly with PwSCI. The discussions were held virtually because we were in the midst of the pandemic. This virtual environment was more challenging, especially for the Delphi Rounds and proceeding with the actual review and decisions regarding the selection of tools and measures. Online surveying was also necessary for our work, and our stakeholders had to be reminded of the importance of completing these and to take time out of their busy schedules. As we wanted to reach a 75% consensus on selected tools or measures, we went back to stakeholders several times and in different ways to get their input and ensure we had adequate response rates. Stakeholders reached the targeted ≥75% consensus needed regarding decisions for selecting tools or selecting and/or developing specific measures/items for one tool [16,30,43]. There was a realization that no one tool could be perfect in measuring all of the experiences relevant to any particular issue or circumstance, and since this tool was intended to be a snapshot, stakeholders accepted this limitation with one tool versus 10 tools. The strength of our work around this was having the involvement of diverse stakeholders including PwSCI who had lived experience as well as others with professional experience to support the decisions made. An additional strength of our tool was that it was essentially co-developed with and by persons with SCI for persons with SCI to be used in evaluating, monitoring, and self-managing their necessary care and support as their health, function, and QOL changed over time. Stakeholders involved in this initiative had entered into unchartered territory, which always has the potential of being unsuccessful, but they were all confident we were trying to create a better tool and process to facilitate improved care, service provision, and follow-up with PwSCI. 

The real test of this tool was planned for the pilot and cognitive reliability testing with PwSCI and other stakeholders, which follows in the last phase of the protocol. With initial testing completed, the tool can then be refined and launched for further validation testing, as well as for testing its utility in the practice setting, with PwSCI taking the results of their tool to meetings or appointments they have with various care and service providers. 

## 5. Conclusions

As it is never an easy task to select key measures for evaluating transitions in care or changes in health, function, and QOL, having a diverse stakeholder group involved, including those with lived experience, is key to the success of the process. This was the case with PwSCI and related stakeholders as part of the CONCENTRIC transitions in care study. In this paper, we presented a protocol for a staged approach to determining the measures/tools needed for PwSCI to evaluate their experiences with transitions in care and changes to health, function, and QOL. We provided SCI stakeholders including PwSCI opportunities to participate in the Delphi Rounds and/or in working groups to critically review and decide on key measures or tools to assess transitions in care and changes in health, function, and QOL. Ten tools that were most commonly used with PwSCI to evaluate or monitor their transitions in care journey were chosen to be simplified or condensed into one tool. The intent was to co-design a unique tool to be used by PwSCI to evaluate their experiences at different times in their transition journey (i.e., at the time of discharge from acute or rehabilitation care and 3, 6, and 12 months or more post-discharge) and use their responses with their care providers to address issues or gaps in their care and support. This new tool has been piloted, refined, and launched for further reliability and validation testing by the population of persons with SCI as part of the last stage of the protocol for tool development. 

## Figures and Tables

**Table 1:** Inclusion criteria for searching published and gray literature, including clinical guidelines or reports.

Surveys/tools or measures reported in published or gray literature studies, or as part of clinical practice guidelines or reports. Tools/measures focused on transitions in/of care, reintegration into the community, and changes in health, function, and QOL as well as related areas of care and self-management. Tools/measures used in relation to persons with chronic neuromuscular or disability or other related conditions, including SCI specifically. Target tools/measures published or cited in the past 15 years. Tools/measures accessible free of charge or have registration or licensing requirements. Tools/measures may or may not be validated or reliability tested with psychometric results for SCI population. Tools/measures accessible for review or have summaries of contents. Tools/measures available in English language.

**Table 2:** Tools identified for measuring outcome domains of transitions in care and changes in health, function, and quality of life.

Outcomes/ Domains	Sub-Outcomes	Tools/Surveys
**Transition in Care**	Knowledge Translation from Stakeholders to Patients; Patient Awareness of Resources after Discharge	Needs and Assessment Checklist (NAC) Patient Continuity of Care Questions (PCCQ long) Care Transition Measure (CTM-15) Partners at Care Transition Measure (PACT—M) Family Caregiver Activation in Transitions (FCAT)
**Changes in Health**	Physical Health	Short Form 12 Health Survey (SF-12) Short Form 36 Health Survey (SF-36) American Spinal Injury Association (ASIA) International Standards for Neurological Classification of Spinal Cord Injury (ISNCSCI) Sickness Impact Profile (SIP68)
	Mental Health	Patient Health Questionnaire-9 (PHQ-9) Generalized Anxiety Disorder Scale (GAD-7) Impact of Event Scale (IES) Body Perception Questionnaire (BPQ)
**Changes in Function**	Instrumental Activities of Daily Living (IADL)	Spinal Cord Independence Measure (SCIMIII) Lawton Instrumental Activities of Daily Living (AIADL) Frenchay Activities Index (FAI) Canadian Occupational Performance Measure (COPM)
	Self-management and ADLs	EQ5D-5L Spinal Cord Independence Measure (SCIM III) COPM
	Community Participation and Reintegration	Reintegration to Normal Living Index (RNLI) Craig Handicap Assessment and Reporting Technique (CHART) COPM Community Integration Questionnaire (CIQ) Community Integration Measure (CIM)
**Changes in Quality of Life**	Quality of Life	Quality of Life Index SCI Version III WHOQOL-BREF (SC-focused version) Satisfaction with Life Scale (SWLS) Perceived Quality of Life Index (QLI) Perceived Quality of Life (PQOL) Global Quality of Life Quality of Life Index (QLI) Life Satisfaction Questionnaire (LISAT-9/-11 Sense of Well-being Index (SWBI) Quality of Life Profile for Adults with Physical Disabilities (QOLP-PD) Quality of Life and Needs Assessment (QOLNA) Personal Wellbeing Index (PWI) SF-36 SF-12 Quality of Well-being Scale QWB) Spinal Cord Injuries Quality of Life—23-item questionnaire (SIQL-23 Patient Reported Impact of Spasticity Measure (PRISM) Leisure Satisfaction Scale (LSS) Leisure Time Physical Activity Questionnaire for People with Spinal Cord Injury (LTPAQ-SCI) Moorong Self-Efficacy Scale (MSES) Comprehensive Quality of Life Scale for Adults v.5 (COMQOL-A5)

**Table 3:** Profile of tools selected by stakeholders for best measuring the four outcomes/domains for persons with SCI (including the number of items per tool and measurement constructs, as well as indicating if tools had or had not been validated by the SCI population).

Outcomes/Domains	Sub-Outcomes	Tools/Surveys (Number of Items/Tool and Measurement Constructs)	Validation for SCI
**Transition in Care**	Knowledge Translation from Stakeholders to Patients; Patient Awareness of Resources after Discharge	**Needs and Assessment Checklist (NAC)**—199 behavioral indicators for assessing patient achievement in nine core areas of rehabilitation.	No
		**Patient Continuity of Care Checklist (PCCQ long)**—41 items for assessing the care received by persons before and after discharge from hospital.	No
**Changes in Health**	Physical Health	**Short Form 36 Health Survey (SF-36)**—36 items related to physical, other health (mental, emotional), and social functioning; one health transition supplementary question.	Yes
		**American Spinal Injury Association (ASIA) International Standards for Neurological Classification of Spinal Cord Injury (ISNCSCI)**—clinician-administered motor and sensory scale that is used to classify the severity of injury to the spinal cord.	Yes
	Mental Health	**Patient Health Questionnaire-9 (PHQ-9)**—9 items for multipurpose screening, diagnosis, monitoring, and measuring severity of depression. **Generalized Anxiety Disorder Scale (GAD-7)**—7 items for screening for generalized anxiety disorder in primary care settings.	No
**Changes in Function**	Instrumental Activities of Daily Living (IADL)	**Spinal Cord Independence Measure (SCIMIII)**—an international version three of a disability scale with 17 items that has been developed specifically for the SCI population in order to assess various ADLs.	Yes
	Self-management and ADLs	**EQ5D-5L**—The EQ-5D-5L health questionnaire has six items that provide a simple descriptive profile and single index value for health status, involving 5 dimensions— mobility, self-care, usual activities, pain/discomfort, and anxiety/depression.	No
		**Spinal Cord Independence Measure (SCIM III)**	Yes
	Community Participation and reintegration	**Reintegration to Normal Living Index (RNLI)**—Self-report questionnaire that assesses an individual’s satisfaction with performance in life activities. Contains 11 items that assess mobility, self-care, daily activity, recreational activity, and family roles.	Used but not validated by SCI population.
**Changes in Quality of Life**	Quality of Life	**Quality of Life Index SCI Version III**—37 items designed to measure subjective quality of life within four domains: Health and functioning, psychological/spiritual, social and economic, and family.	Used but not validated by SCI population.
		**World Health Organization Quality of Life-BREF (WHOQOL-BREF-SCI focused version)**—26 items assessing four domains—physical health, psychological, social relationships, and environment	Yes

## Data Availability

Data gathered through the modified Delphi Rounds and working groups can be made available on request.

## Supplementary Materials

The following supporting information can be downloaded at: https://www.mdpi.com/article/10.3390/healthcare12050527/s1, Figure S1: Geography of Alberta as part of Canada, and locations of Edmonton, Calgary and two spoke sites of Slave Lake and Lethbridge; Table S1: Mapping of Identified Themes and Measurement Indicators/Items for CONCENTRIC Outcomes/Domains (Transitions in Care, and Changes in Health, Function and Quality of Life) and by Categories (Before/During Discharge’ and ‘After Discharge -Self & Home/Community Transition’); File S1: Co-developed Tool for Self-Evaluation by Person with Spinal Cord Injury (SCI).
