# Supplementary material for: Measures for Persons with Spinal Cord Injury to Monitor Their Transitions in Care, Health, Function, and Quality of Life Experiences and Needs: A Protocol for Co-Developing a Self-Evaluation Tool
Source: Healthcare (Basel). 2024 Feb 23;12(5):527. doi: 10.3390/healthcare12050527 (PMC10930772; doi:10.3390/healthcare12050527)

**Supplementary File S1: Co-developed Tool for Self-Evaluation by Person with Spinal Cord Injury (SCI)**


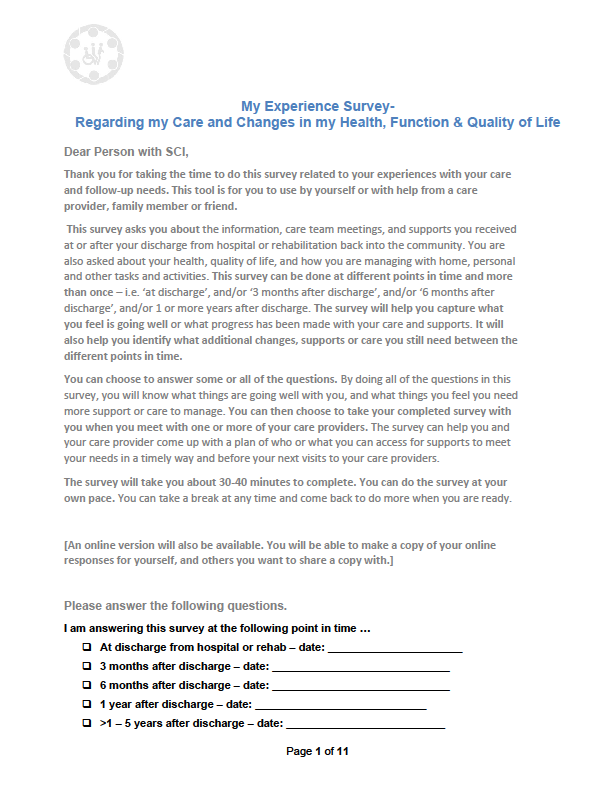


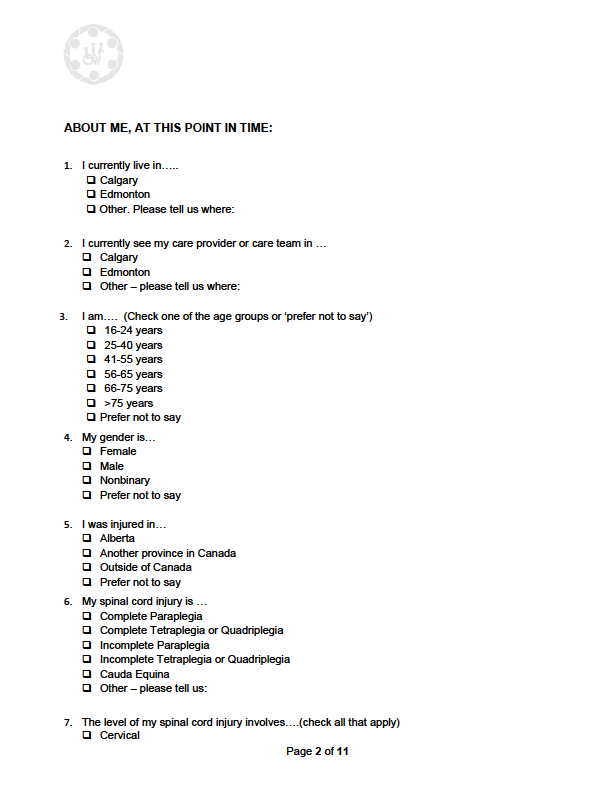


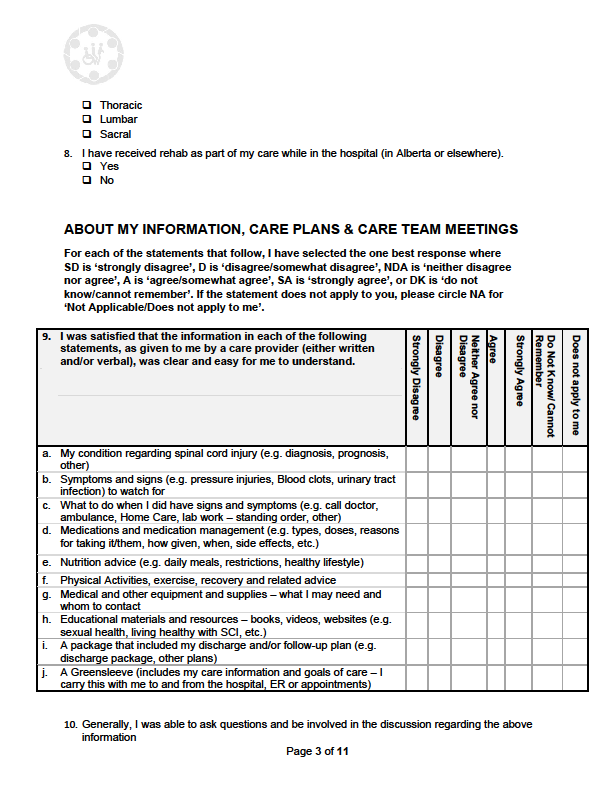


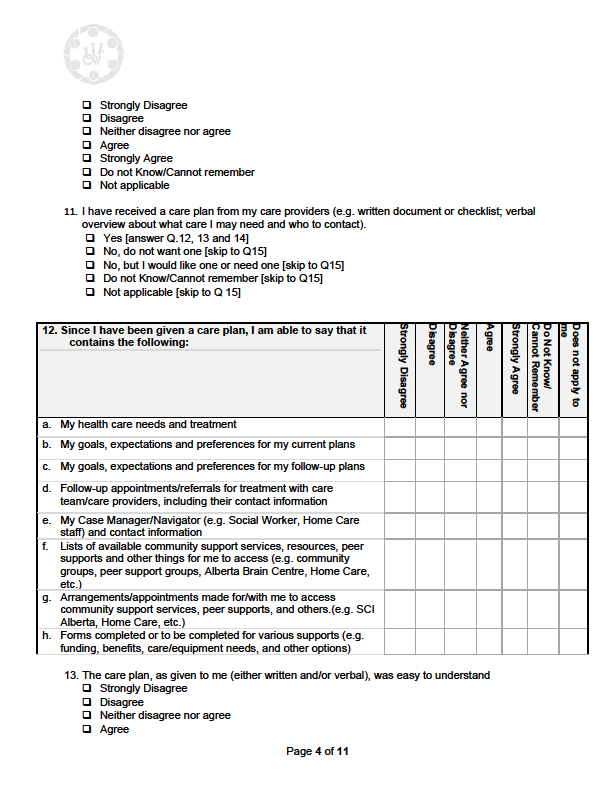


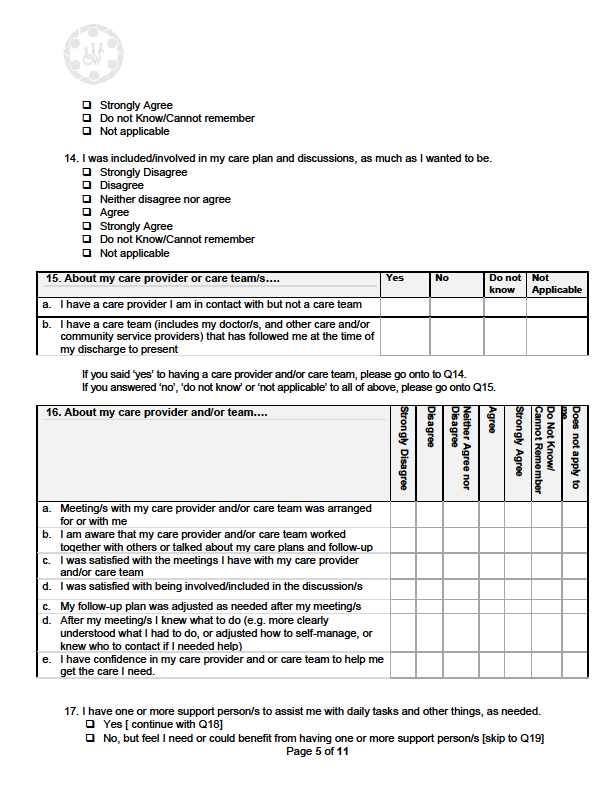


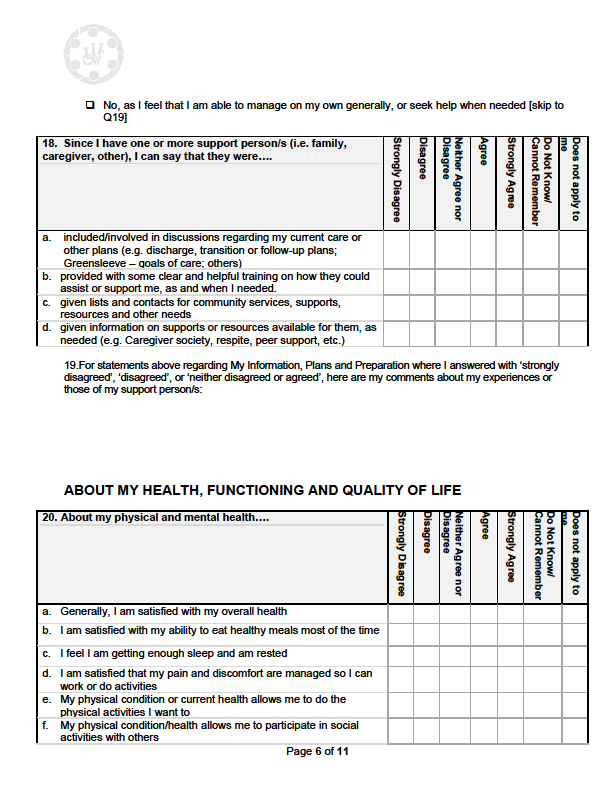


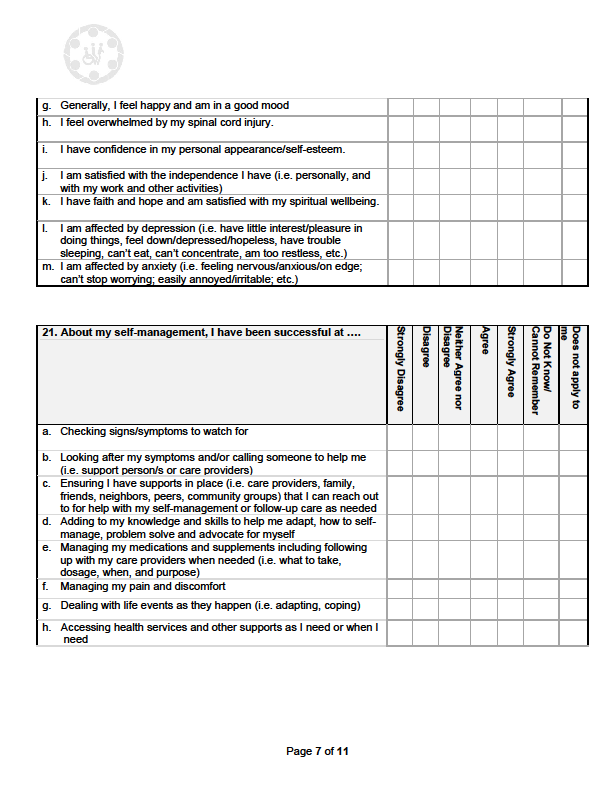


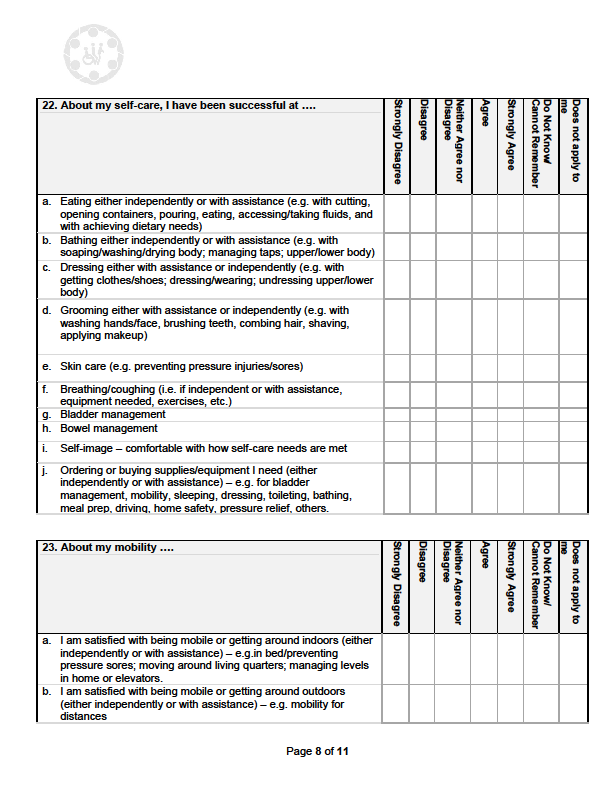


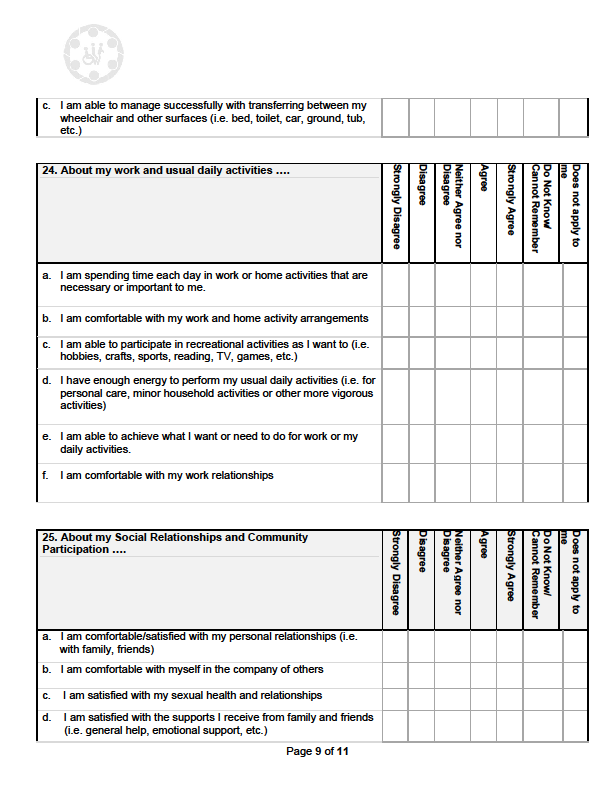


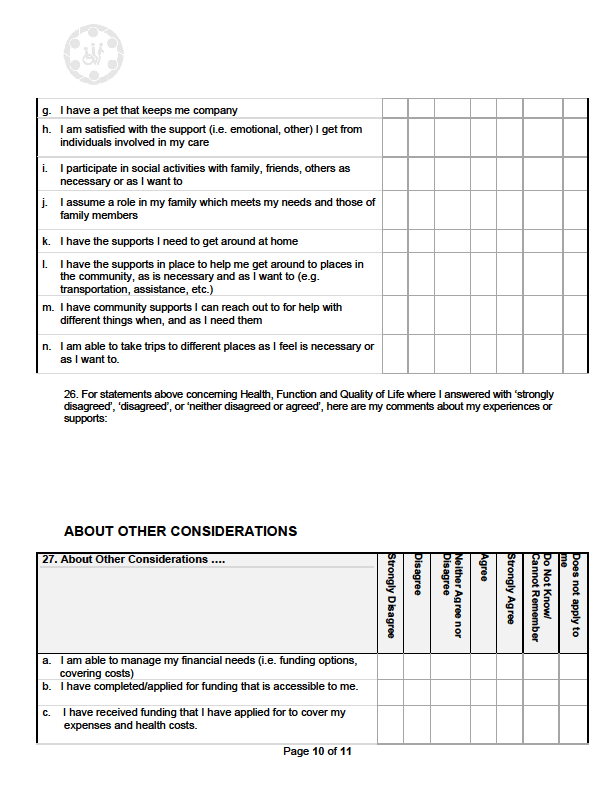


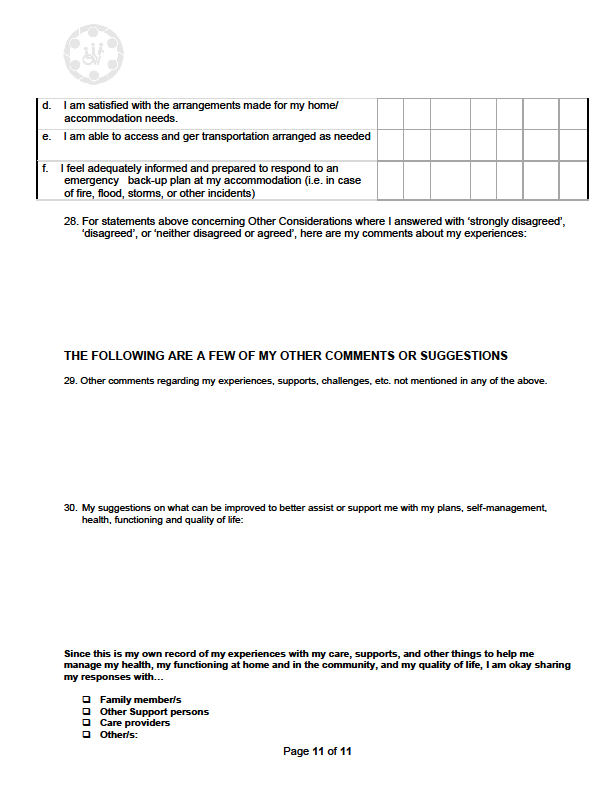

Supplement: Supplementary file 1 [file healthcare-12-00527-s001.zip › Supplementary File S1- Person with SCI self-evaluation tool.docx]
