# Supplementary material for: Measures for Persons with Spinal Cord Injury to Monitor Their Transitions in Care, Health, Function, and Quality of Life Experiences and Needs: A Protocol for Co-Developing a Self-Evaluation Tool
Source: Healthcare (Basel). 2024 Feb 23;12(5):527. doi: 10.3390/healthcare12050527 (PMC10930772; doi:10.3390/healthcare12050527)

**Supplementary Table S1: Mapping of Identified Themes and Measurement Indicators/Items for CONCENTRIC Outcomes/Domains (Transitions in Care, and Changes in Health, Function and Quality of Life) and by Categories (Before/During Discharge’ and ‘After Discharge -Self & Home/Community Transition’)**


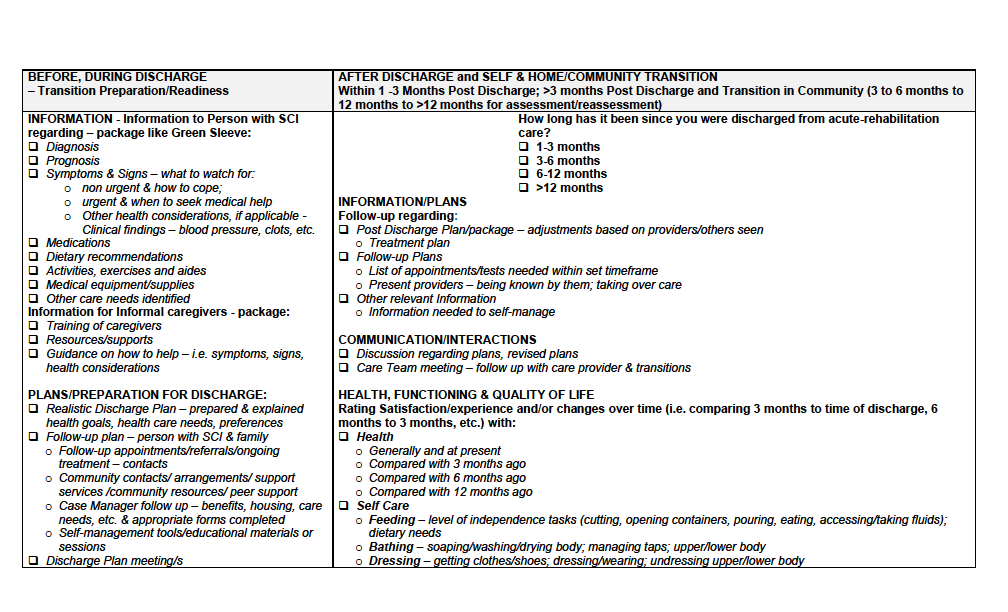


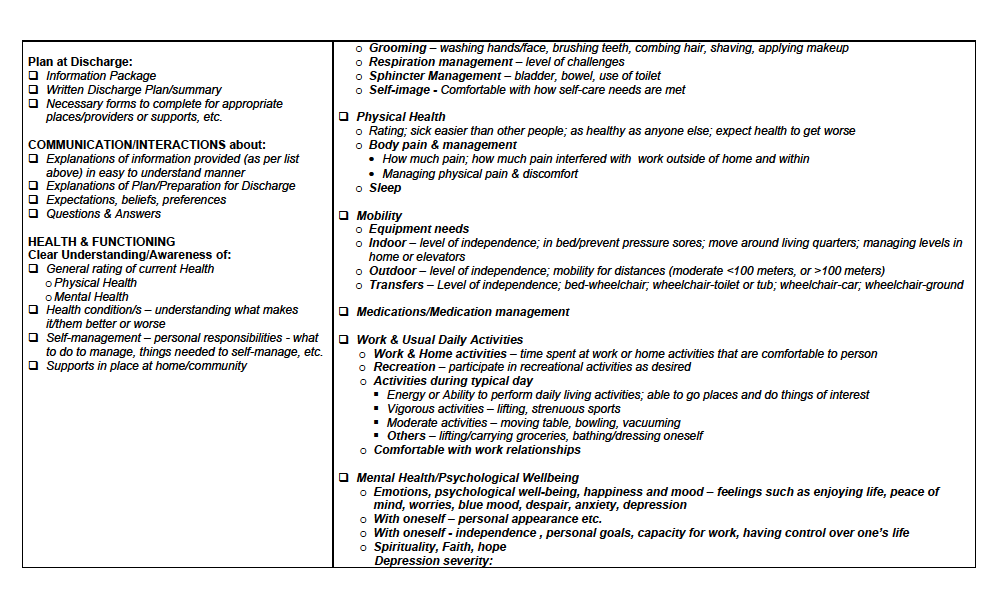


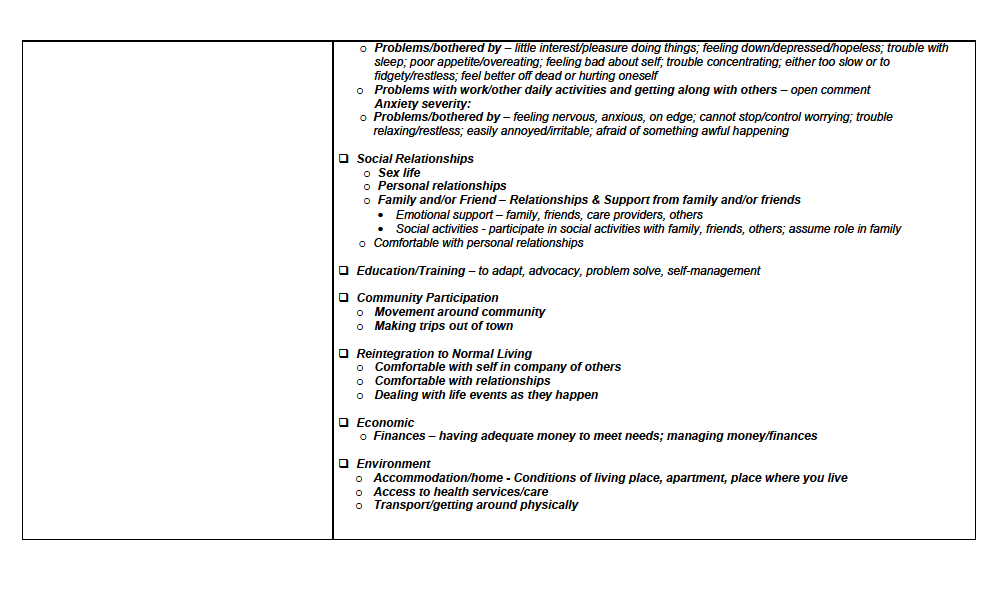

Supplement: Supplementary file 1 [file healthcare-12-00527-s001.zip › Supplementary Table S1 final.docx]
